# The prognostic and predictive roles of plasma C‐reactive protein and PD‐L1 in non‐small cell lung cancer

**DOI:** 10.1002/cam4.6262

**Published:** 2023-06-16

**Authors:** Saara Kuusisalo, Antti Tikkanen, Elisa Lappi‐Blanco, Timo Väisänen, Aija Knuuttila, Satu Tiainen, Jarkko Ahvonen, Sanna Iivanainen, Jussi P. Koivunen

**Affiliations:** ^1^ Department of Medical Oncology and Radiotherapy and Medical Research Center Oulu Oulu University Hospital and University of Oulu Oulu Finland; ^2^ Department of Pathology, Oulu University Hospital and Department of Pathology, Cancer and Translational Medicine Research Unit University of Oulu Oulu Finland; ^3^ Department of Pulmonary Medicine, Heart and Lung Center and Cancer Center Helsinki University Hospital and University of Helsinki Helsinki Finland; ^4^ Cancer Center Kuopio University Hospital Kuopio Finland; ^5^ Faculty of Medicine and Health Technology, Tampere University and Tays Cancer Center, Department of Oncology Tampere University Hospital Tampere Finland

**Keywords:** CRP, ICI, NSCLC, PD‐L1, predictive, prognostic

## Abstract

**Background:**

Anti‐PD‐(L)1 agents have revolutionized the treatment paradigms of non‐small cell lung cancer (NSCLC), while predictive biomarkers are limited. It has been previously shown that systemic inflammation, indicated by elevated C‐reactive protein (CRP) level, is associated with a poor prognosis in anti‐PD‐(L)1 treated. The aim of the study was to analyze the prognostic and predictive value of CRP in addition to traditional prognostic and predictive markers and tumor PD‐L1 score.

**Methods:**

We identified all NSCLC patients (*n* = 329) who had undergone PD‐L1 tumor proportion score (TPS) analysis at Oulu University Hospital 2015–22. CRP levels, treatment history, immune checkpoint inhibitor (ICI) therapy details, and survival were collected. The patients were categorized based on CRP levels (≤10 vs. >10) and PD‐L1 TPS scores (<50 vs. ≥50).

**Results:**

In the whole cohort (*n* = 329), CRP level of ≤10 mg/L was associated with improved survival in univariate (HR 0.30, Cl 95% 0.22–0.41) and multivariate analyzes (HR 0.44, CI 95% 0.28–0.68). With ICI treated (*n* = 70), both CRP of ≤10 and PD‐L1 TPS of ≥50 were associated with improved progression‐free survival (PFS) in univariate (HR 0.51, CI 95% 0.27–0.96; HR 0.54, CI 95% 0.28–1.02) and multivariate (HR 0.48, CI 95% 0.26–0.90; HR 0.50, CI 95% 0.26–0.95) analyzes. The combination (PD‐L1 TPS ≥50 and CRP >10) carried a high negative predictive value with a median PFS of 4.11 months (CI 95% 0.00–9.63), which was similar to patients with low PD‐L1 (4.11 months, CI 95% 2.61–5.60).

**Conclusions:**

Adding plasma CRP levels to PD‐L1 TPS significantly increased the predictive value of sole PD‐L1. Furthermore, patients with high CRP beard little benefit from anti‐PD‐(L)1 therapies independent of PD‐L1 score. The study highlights the combined evaluation of plasma CRP and PD‐L1 TPS as a negative predictive marker for ICI therapies.

## Graphical abstract

The study shows that patients with high C‐reactive protein (CRP) beard little benefit from programmed death‐1 receptor (PD‐1) and its ligand (PD‐(L)1) therapies independent of PD‐L1 score and highlights the value of combined evaluation of plasma CRP and PD‐L1 TPS as a negative predictive marker for ICIs.

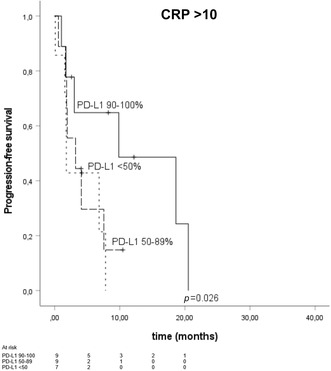

## 1 INTRODUCTION

Immune checkpoint inhibitor (ICI) therapies have transformed cancer care in multiple tumor types, both in advanced and adjuvant settings.^1^, ^2^, ^3^, ^4^, ^5^, ^6^, ^7^, ^8^, ^9^, ^10^, ^11^, ^12^ Programmed death‐1 receptor (PD‐1) and its ligand (PD‐L1) are one of the most important mediators of immune checkpoints and their targeting can reactivate antitumor immunity.^13^, ^14^ Monoclonal antibodies targeting the PD‐1/PD‐L1 axis have demonstrated clinically significant antitumor activity and less frequent treatment‐related adverse effects compared to chemotherapy.^1^

The development of anti‐PD‐(L)1 agents has revolutionized the treatment paradigms of non‐small cell lung cancer (NSCLC) with long lasting therapy responses and clinically meaningful overall survival benefit.^4^, ^5^, ^6^, ^7^, ^8^, ^15^ In advanced NSCLC, the first line treatment with anti‐PD‐(L)1 agents is recommended in patients with ≥50% PD‐L1 tumor proportion score (TPS) and in combination with chemotherapy with lower PD‐L1 scores.^7^, ^15^, ^16^, ^17^ Furthermore, later line ICI treatment is applicable regardless of PD‐L1 scores.^4^, ^5^, ^6^, ^8^ The benefit from anti‐PD‐(L)1 agents is associated to PD‐L1 TPS and patients with higher score have an increased likelihood of responding to the therapy which is especially evident with a TPS of ≥50%.^6^

Objective response rates (ORR) in patients receiving anti‐PD‐(L)1 inhibitor therapy for advanced NSCLC are around 20% while most patients fail to respond. Despite several studies investigating predictive markers for ICI therapies in NSCLC, there is still a lack of robust biomarkers.^14^, ^18^, ^19^ PD‐L1 TPS of ≥50% was first shown to display a higher ORR in the KEYNOTE‐001 and KEYNOTE‐010 studies compared to those with 50% or lower PD‐L1 TPS in NSCLC. PD‐L1 TPS of ≥50% has been well adopted as a predictive marker in the clinical setting but its prognostic role remains controversial.^6^, ^20^

Systemic, cancer‐related inflammation has been associated with a poorer prognosis and is proposed to contribute to the tumor immunoresistance.^21^ Many investigators have linked systemic inflammation to ICI therapy resistance. It is debatable whether systemic inflammation is directly linked to ICI resistance or is it reflecting an advanced malignancy in which the cancer itself induces immunosuppression. There is supporting evidence that systemic inflammation is linked to immunosuppressive tumor microenvironment (TME). Molecular mechanisms of the immunosuppressive TME are only partly described and may involve altered metabolism, regulatory T‐cells, macrophage polarization, and IL‐6 production all leading to impaired T‐cell function.^22^, ^23^

The ratios of monocytes and neutrophils against lymphocytes together with lactate dehydrogenase and CRP levels have been utilized as markers of systemic inflammation. Among the biomarkers only CRP and neutrophil‐lymphocyte ratio (NLR) have exhibited predictive capability for ICI therapies in NSCLC.^24^, ^25^ We have previously shown that baseline CRP, with a cut‐off of 10 mg/L, is a very strong indicator for prognosis in ICI treated multiple advanced cancer types and the identification and validation of the CRP cut‐off is explained in detail in our previous publication.^26^ In brief, ROC curve was calculated to define the optimal cut‐off point of CRP in the discovery cohort and this was validated in an independent cohort. Furthermore, another research group has identified the same cut‐off for CRP for ICI treated advanced melanomas. Interestingly, they also showed in vitro that CRP suppressed T cell immunity and function, and levels above 10 mg/L inhibited T cell proliferation and altered T cell signaling, suggesting that CRP can even directly affect the earliest steps in T cell signaling and activation.^27^

In the current work, we investigated the prognostic and predictive values of PD‐L1 TPS and baseline plasma CRP levels, and their combination. Our main hypothesis was that incorporation of CRP levels to prognostication would improve the predictive value of using sole tumor PD‐L1 TPS.

## 2 METHODS

Data collection was carried out according to national legislation and under a permit from the medical director of Oulu University Hospital (study no. 299/2016). Pseudonymization was carried out before data analysis. Informed consent was not sought due to the register nature of the study.

All patients who had been analyzed for tumor PD‐L1 TPS % at Oulu University Hospital 1/2015–9/2022 were identified from the pathology records. All the cases were manually reviewed and patients meeting the study inclusion and fulfilling no exclusion criteria were included the cohort. The inclusion criteria were PD‐L1 analyzed from histological sample with existing TPS score, NSCLC diagnosis, and complete treatment history for NSCLC available at electronic patient records. The exclusion criteria were non‐existing PD‐L1 TPS score, other diagnosis than NSCLC, other malignancy within 3 years from NSCLC diagnosis, or missing information on the overall treatment history.

Data collected from the electronic patient records included date of birth, gender, ECOG score at the time of noncurative stage, smoking pack years, date of diagnosis, primary stage, histology, tumor PD‐L1 TPS score, existence of EGFR/ALK/ROS1 alterations, primary treatment, ICI therapy, the treatment line of ICI therapy, single agent ICI or combination, the first and last date of ICI therapy, PD date on ICI therapy or the last day of follow‐up, and date of death or the last day of follow‐up. The analysis of the ICI treated was limited only to the patients who received ICI as a part of noncurative treatment regimen.

PD‐L1 TPS score was analyzed using E1L3N antibody (Cell Signaling Technology) with Leica Bond Autostainer at Department of Pathology in Oulu University Hospital. The applied method has scored perfect in NordiQC and European Society of Pathology (ESP) external quality assessment rounds. The peripheral blood CRP levels were measured in a FINAS accredited (Nordlab, SFS‐EN ISO 15189) laboratory with immunoturbidimetric test. CRP values taken within +/− 4 weeks from tumor sampling used for PD‐L1 analysis were recorded. If a patient underwent surgical resection for lung cancer, only preoperative CRP values were registered. If blood samples were taken during clinically confirmed acute infection based on EHR (which includes details of medication, such as antibiotics), another preinfection or postinfection value was chosen which timely lined with the closest to the tumor sampling used for PD‐L1 analysis to control the confounding effect of possible acute infection.

Overall survival (OS) was calculated from the date of diagnosis to the date of death or end of follow‐up. Progression free‐survival (PFS) and overall survival on IO (OS IO) were calculated from the first date of ICI treatment to the documented tumor progression, death, or end of follow‐up (PFS) or to death or end of follow‐up (OS IO). Tumor progression and/or death were counted as an event. IBM SPSS Statistics 27.00.00 for Windows was applied for statistical analysis. Survival was analyzed by using the Kaplan–Meier and Cox regression methods with 95% confidence intervals. Pearson's chi‐square test was used for analyzing the differences between the groups.

## 3 RESULTS

### 3.1 Demographics

A total of 329 patients with NSCLC analyzed for tumor PD‐L1 TPS at Oulu University Hospital 2015–2022 were included in the study. The ethnicity of the study population is ~99% Caucasian. The median (m) age of the patients was 70 years, and the majority of the patients (60.5%) were male. The cohort included patients with NSCLC of varying histologies, such as adenocarcinoma (*n* = 187, 56.8%), and squamous cell carcinoma (*n* = 86, 26.1%); the rest of the cohort had other histologies (*n* = 56, 17.0%). At the time of diagnosis, 169 (51.5%) patients had Stage IV, 75 (22.8%) Stage III, 36 (10.9%) Stage II, and 49 (14.9%) Stage I disease. CRP levels were available from a total of 306 (93.0%) patients, of whom 152 (46.2%) had CRP of ≤10 and 154 (48.8%) CRP of >10. Most of the patients (*n* = 203, 61.7%) had PD‐L1 TPS of <50%. Therapeutic targetable genetic alterations, such as epidermal growth factor receptor (EGFR), anaplastic lymphoma kinase (ALK), and c‐ros oncogene 1 (ROS1), were detected in 42 (12.8%) patients. 101 (30.7%) patients received surgery, 24 (7.3%) curative radiotherapy, 125 (38.0%) systemic noncurative treatment, and 79 (24.0%) palliative radiotherapy or best supportive care as primary treatment (Table 1).

**TABLE 1:** Patient demographics.

	*n* (%)
All	329 (100)
Age (median), years	70
Sex	
Total	329 (100)
Male	199 (60.5)
Female	130 (39.5)
Smoking pack years	
Total	310 (94.2)
<20	88 (26.7)
≥20	222 (67.5)
Stage at diagnosis	
Total	329 (100)
I	49 (14.9)
II	36 (10.9)
III	75 (22.8)
IV	169 (51.5)
CRP	
Total	306 (93.0)
≤10	152 (46.2)
>10	154 (48.8)
PD‐L1	
Total	329 (100)
<50	203 (61.7)
≥50	126 (38.3)
Tumor histology	
Total	329 (100)
Adenocarcinoma	187 (56.8)
Squamous cell carcinoma	86 (26.1)
Other	56 (17.0)
Targetable genetic alteration	
Total	42 (12.8)
EGFR	34 (10.3)
ALK	4 (1.2)
ROS1	4 (1.2)
ECOG performance status (at non‐curative stage)	
Total	241 (73.3)
0	37 (11.2)
1	103 (31.3)
2	67 (20.4)
3	26 (7.9)
4	8 (2.4)
Primary treatment	
Total	329 (100)
Surgery	101 (30.7)
Curative radiotherapy	24 (7.3)
Systemic non‐curative treatment	125 (38.0)
Palliative radiotherapy/BSC	79 (24.0)

*Note*: Values are presented as *n* (%) unless indicated otherwise.

Abbreviations: BSC, best supportive care; CRP, C‐reactive protein; PD‐L1, programmed death‐1 receptor (PD‐1) and its ligand.

Next, we evaluated whether CRP levels (≤10 vs. >10 mg/L) associated with known baseline prognostic factors. High CRP levels correlated with male gender, ≥20 pack years, Stage III‐IV, histology other than adenocarcinoma, and ECOG score ≥2. We did not observe any association with CRP and PD‐L1 TPS score (Table 2).

**TABLE 2:** Patient demographics by CRP levels.

	CRP ≤10 *n* (%)	CRP >10 *n* (%)	*p*‐Value*
Sex			
Male	79 (42.2)	108 (57.8)	0.001
Female	73 (61.3)	46 (38.7)	
Smoking pack years			
<20	57 (67.9)	27 (32.1)	<0.001
≥20	91 (43.5)	118 (56.5)	
Stage at diagnosis			
I‐II	63 (77.8)	18 (22.2)	<0.001
III‐IV	89 (39.6)	136 (60.4)	
PD‐L1			
<50	102 (53.7)	88 (46.3)	NS
≥50	50 (43.1)	66 (56.9)	
Tumor histology			
Adenocarcinoma	110 (64.0)	62 (36.0)	<0.001
Other	41 (30.8)	92 (69.2)	
ECOG performance status (at non‐curative stage)			
0–1	70 (55.6)	56 (44.4)	<0.001
≥2	25 (26.0)	71 (74.0)	

* Chi‐square test

Abbreviation: CRP, C‐reactive protein; PD‐L1, programmed death‐1 receptor (PD‐1) and its ligand.

### 3.2 Prognostic values of CRP and PD‐L1 TPS

**FIGURE 1:**
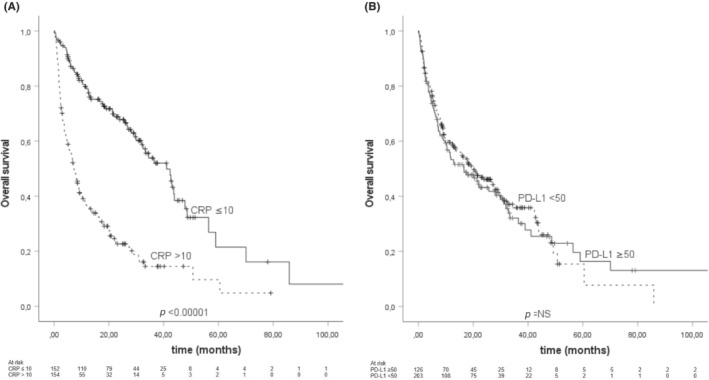
Kaplan–Meier analysis for overall survival according to (A) peripheral blood C‐reactive protein (CRP) level (B) Programmed death‐1 receptor (PD‐1) and its ligand (PD‐L1) tumor proportion score (TPS) score in the whole study population. Crosses indicate censored events.

OS in the whole cohort was analyzed separately according to the patient's peripheral blood CRP levels (taken within +/− 4 weeks from tumor sampling used for PD‐L1 analysis) and tumor PD‐L1 TPS score (Figure 1A,B). CRP levels were divided into two categories of >10 and ≤10, and PD‐L1 TPS scores to ≥50 and <50. The mOS for the whole cohort was 18.7 months (CI 95% 12.8–24.5). In univariate analysis for OS, CRP level of ≤10 was associated with improved OS (HR 0.30, Cl 95% 0.22–0.41) while PD‐L1 TPS of ≥50 showed no association to OS (HR 1.05, CI 95% 0.79–1.49). In multivariate analysis, including sex, pack‐years, primary stage, histology, and EGFR‐ALK‐ROS1 status, the association between improved OS and CRP level of ≤10 was retained (HR 0.44, CI 95% 0.28–0.68). Interestingly, the only other prognostic marker retaining significance in multivariate analysis was stage (0.43, CI 95% 0.23–0.81) (Table 3).

**TABLE 3:** Univariate and multivariate analysis for overall survival (OS).

	Univariate		Multivariate	
	HR	CI (95%)	HR	CI (95%)
Sex				
Male vs. Female	1.552	1.153–2.088	1.146	0.777–1.690
Pack years				
≥20 vs. <20	1.536	1.092–2.162	1.325	0.828–2.120
Primary stage				
I‐II vs. III‐IV	0.194	0.126–0.299	0.428	0.226–0.810
Histology				
Adenocarcinoma vs. Other	0.611	0.463–0.807	0.808	0.523–1.246
EGFR‐ALK‐ROS1				
Positive vs. negative	0.559	0.357–0.877	0.471	0.456–1.438
CRP				
≤10 vs. >10	0.304	0.223–0.414	0.441	0.284–0.683
PD‐L1				
≥50 vs. <50	1.048	0.789–1.485	0.961	0.662–1.396

Abbreviation: PD‐L1, programmed death‐1 receptor (PD‐1) and its ligand.

### 3.3 Predictive values of CRP and PD‐L1 TPS for ICI treated patients

In the cohort, 70 patients were treated with ICI therapies in non‐curative intent. Majority of the patients received ICI therapy as a single agent therapy (*n* = 59, 84%), while the rest (*n* = 11, 16%) as a combination treatment with chemotherapy. ICI was given as a first line treatment to 54 patients (77%), while 16 (23%) received the therapy in the 2nd or 3rd line setting. CRP levels were available for a total of 67 (95.7%) ICI treated patients, of whom 41 (61.1%) had CRP of ≤10, and 26 (38.8%) CRP of >10. The majority of ICI‐treated patients (*n* = 48, 68.6%) had PD‐L1 TPS of ≥50% (Table 4).

**TABLE 4:** Patient demographics of PD‐(L)1 treated.

	*n* (%)
All	70 (100)
Age (median), years	68
Sex	
Total	70 (100)
Male	51 (72.9)
Female	19 (27.1)
Smoking Pack Years	
Total	67 (95.7)
<20	11 (16.4)
≥20	56 (83.6)
Stage at diagnosis	
Total	70 (100)
I‐II	10 (14.3)
III‐IV	60 (85.7)
CRP	
Total	67 (95.7)
≤10	41 (61.2)
>10	26 (38.8)
PD‐L1 TPS	
Total	70 (100)
<50	22 (31.4)
≥50	48 (68.6)
Line of PD‐(L)1 treatment	
Total	70 (100)
First line	54 (77.1)
Second or later line	16 (22.9)
Treatment schema	
Total	70 (100)
Single agent	59 (84.3)
Combination with chemo	11 (15.7)
Stage at PD‐(L)1 treatment	
Total	70 (100)
IV	44 (62.9)
III	25 (35.7)
II	1 (1.4)

Abbreviations: PD‐L1, programmed death‐1 receptor (PD‐1) and its ligand; TPS, tumor proportion score.

PFS on ICI therapy was analyzed according to the patient's CRP and PD‐L1 TPS score. The mPFS for the whole ICI cohort was 6.8 months (CI 95% 3.7–10.0). In univariate analysis for PFS, CRP level of ≤10 (HR 0.51, CI 95% 0.27–0.95) was associated with improved PFS while PD‐L1 TPS of ≥50 showed tendency for superior PFS (HR 0.54, CI 95% 0.29–1.01). In multivariate analysis including CRP and PD‐L1 score, improved PFS was seen with both CRP level of ≤10 (HR 0.48, CI 95% 0.26–0.90) and PL‐L1 TPS of ≥50 (HR 0.50, CI 95% 0.26–0.95) (Table 4). We also tested other variables (sex, age, pack year, histology, primary stage, and the line of ICI therapy) in univariate analysis, and none of them carried significant prognostic value (Table 5).

**TABLE 5:** Univariate and multivariate analysis for PFS (IO) and OS (IO).

	Univariate		Multivariate	
	HR	CI (95%)	HR	CI (95%)
*PFS (IO)*				
Sex				
Male vs. Female	1.370	0.719–2.609		
Age				
<65y vs. ≥65y	0.701	0.368–1.333		
Pack years				
≥20 vs. <20	0.707	0.310–1.612		
Primary stage				
I‐II vs. III‐IV	0.628	0.246–1.601		
Histology				
Adenocarcinoma vs. Other	0.450	0.430–1.454		
Line of therapy				
First vs. Later	0.684	0.351–1.331		
CRP				
≤10 vs. >10	0.449	0.227–0.886	0.435	0.220–0.862
PD‐L1 TPS				
≥50 vs. <50	0.485	0.250–0.942	0.464	0.235–0.917
*OS (IO)*				
Sex				
Male vs. Female	1.547	0.725–3.301		
Age				
<65y vs. ≥65y	0.859	0.422–1.750		
Pack years				
≥20 vs. <20	0.549	0.222–1.354		
Primary stage				
I‐II vs. III‐IV	0.808	0.314–2.081		
Histology				
Adenocarcinoma vs. Other	0.681	0.347–1.337		
Line of therapy				
First vs. Later	0.626	0.307–1.276		
CRP				
≤10 vs. >10	0.479	0.225–1.021	0.536	0.248–1.159
PD‐L1 TPS				
≥50 vs. <50	0.385	0.180–0.825	0.387	0.175–0.875

Abbreviations: PD‐L1, programmed death‐1 receptor (PD‐1) and its ligand; OS, overall survival; PFS, progression‐free survival; TPS, tumor proportion score.

**FIGURE 2:**
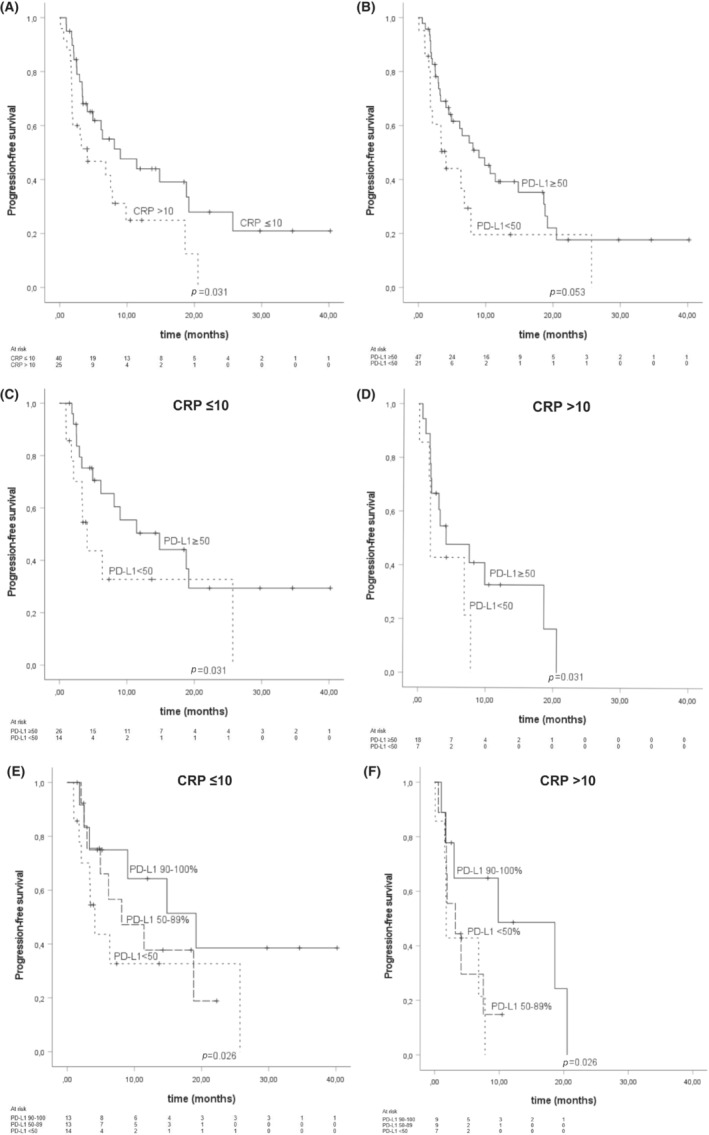
Kaplan–Meier analysis for progression‐free survival according to (A) peripheral blood C‐reactive protein (CRP) level, (B) Programmed death‐1 receptor (PD‐1) and its ligand (PD‐L1) tumor proportion score (TPS) score, (C) PD‐L1 TPS score in patients with peripheral blood CRP ≤ 10, and (D) PD‐L1 TPS score in patients with peripheral blood CRP > 10, (E) PD‐L1 TPS divided in the three categories in patients with CRP ≤10, (F) PD‐L1 TPS divided in the three categories in patients with CRP > 10. Crosses indicate censored events.

The mPFS for patients with CRP ≤10 was 9.0 months (CI 95% 0.99–17.02), while for patients with CRP >10 it was 4.11 months (CI 95% 0.00–9.49). There was also a difference in mPFS between patients with PD‐L1 ≥ 50 (9.00 months, CI 95% 4.42–13.59) and PD‐L1 < 50 (4.11 months, CI 95% 2.62–5.60) (Figure 2A,B). When CRP levels were ≤ 10, the mPFS for patients with PD‐L1 ≥ 50 was 14.85 months (CI 95% 3.56–26.14), while the mPFS for patients with CRP >10 and PD‐L1 ≥ 50 was 4.11 months (CI 95% 0.00–9.63) which near to the observed in PD‐L1 < 50 patients (1.81 months, CI 95% 1.72–1.89) (Figure 2C,D). We also carried out a sub‐classification of PD‐L1 score into three categories (0–49, 50–89, and 90–100%). According to PD‐L1 score, PFS clearly separated to three categories in CRP ≤10 patients. Conversely, in CRP >10 patients, PD‐L1 90–100% patients separated from the other groups while PD‐L1 0–49% and 50–89% had very similar PFS (Figure 2E,F).

Overall survival while on ICI therapy (OS (IO)) was also analyzed according to CRP levels and PD‐L1 TPS score. The median OS (IO) for the whole cohort was 14.4 months (CI 95% 5.99–22.86). In univariate analysis for OS (IO), PD‐L1 TPS of ≥50 was associated with improved OS (IO) (HR 0.40, CI 95% 0.20–0.81), while CRP level of ≤10 showed no statistically significant association with OS (IO) (HR 0.54, CI 95% 0.27–1.08). In multivariate analysis with PD‐L1 score and CRP, the association between improved OS (IO) and PD‐L1 TPS of ≥50 was retained (HR 0.38, CI 95% 0.19–0.78) (Table 5).

## 4 DISCUSSION

While ICIs have changed the treatment landscape of multiple advanced cancers, only minority of the patients benefit from the therapies. Resistance to immunotherapy is not fully understood, and even though the distinction to primary and secondary or acquired resistance is relevant in clinical trial context, it cannot be used to guide treatment selection. Neither offers it any mechanistic insight for the development of more effective therapies or biomarkers. Multiple preclinical and early stage clinical trials are ongoing to identify novel therapeutic targets and/or agents to improve the clinical value of immunotherapies.^28^, ^29^, ^30^

Cancer immunology is a very complex entity, thus, only suboptimal predictive markers for ICI therapy benefit have been characterized.^31^ Pretreatment tumor PD‐L1 expression is the most widely applied biomarker in multiple tumor types. High PD‐L1 expression is a valid ICI‐indication‐bound biomarker in some cancers such as NSCLC, and head and neck squamous cell cancers.^6^, ^15^, ^18^, ^32^ However, both sensitivity and specificity of tumor PD‐L1 expression as a predictive marker for ICI therapies are low, while the prognostic nature of PD‐L1 TPS is inadequately characterized.

Systemic inflammation indicated by factors such as elevated CRP or NLR have been linked to poor survival in numerous cancers such as NSCLC.^25^, ^33^ Furthermore, many studies have linked systemic inflammation to adverse prognosis on cancer patients treated with ICIs.^34^, ^35^, ^36^, ^37^, ^38^ We have previously shown with two independent cohorts that CRP levels >10 mg/L are linked to poor PFS and OS in ICI treated cancer patients and the likelihood of treatment benefit in these patients is very low (mPFS 2.0 months).^26^ Since the obtainability of tumor biopsies can be challenging especially in lung cancers, biomarkers analyzable from peripheral blood could be more feasible.

As in previous studies, we observed that high CRP associated with known poor prognostic factors and inferior survival. CRP had strong independent prognostic value in multivariate analysis even surpassing known prognostic factors. In addition, our study further highlighted the prognostic and predictive values of tumor PD‐L1 score. PD‐L1 has been shown to be a predictive marker for ICI therapies, however, previous studies have shown incongruous results on the prognostic role of PD‐L1 expression.^39^ It is essential to realize that the variety of results and different prognostic impact of PD‐L1 in specific subgroups is influenced by the testing method itself (e.g., different antibodies and thresholds) and individual patient‐related characteristics, such as ethnicity or tumor histology. For instance, different proportions of PD‐L1‐positive tumors for distinct histological NSCLC subtypes have been reported.^40^, ^41^, ^42^, ^43^ In our study, high PD‐L1 TPS score carried both prognostic and predictive value in the ICI treated patients while in the whole cohort, including patients treated in curative settings, only the association between improved OS and CRP level of ≤10 was retained.

To our knowledge, our study is the first to use pretreatment tumor PD‐L1 score and circulating CRP levels as a combined biomarker for ICI benefit. According to the results, both CRP and PD‐L1 bare independent predictive value for ICI therapies (PFS), and PD‐L1 also for OS. More importantly, the benefit from ICI therapies in high PD‐L1 score (≥ 50%) is driven mainly by patients with low CRP levels (≤10 mg/L), since the mPFS for the patients with CRP >10 and PD‐L1 ≥ 50 was 4.11 months, which is similar than with patients with PD‐L1 < 50 (mPFS 4.11 months). The patients with very high PD‐L1 score (90–100%), bared PFS benefit from PD‐L1 therapies regardless of CRP value, while with lower PD‐L1 TPS score, the benefit was only seen with CRP low patients.

Only limited ICI benefit was seen with high CRP levels (>10 mg/L) that was analogous to the outcomes of patients with low PD‐L1 scores. Our results highlighted the combined value of PD‐L1 TPS and CRP as predictive biomarker for ICI therapies. It is unknown, how incorporating CRP levels to PD‐L1 increases the predictive value of the marker. Immunohistochemical staining for protein expression, such as PD‐L1, is notoriously challenging due to the tissue heterogeneity,^44^ thus, analyzing CRP could even out the limitations of tumor‐based analyzes. Systemic inflammation specified by elevated CRP levels may reflect a direct effect of inflammation leading to an immunosuppressive TME or merely indicating an advanced cancer with systemic manifestation such as anorexia, cachexia, and anemia resulting immunosuppression.^23^ If the first is considered as the main mechanism of immunosuppression, one can hypothesize that high CRP could indicate a TME in which anti‐PD‐(L)1 antibodies are unable to initiate antitumor immunity.

Our study has some obvious limitations. The number of subjects, especially in the ICI treated group, is limited, and this adds uncertainty to the study results. Retrospective design has its pitfalls and prospective design is often required to minimize the bias and estimate the real predictive value of a biomarker. In the ICI treated cohort, most of the patients received single agent therapy and we cannot conclude whether our findings do hold on to patients treated with ICI‐chemotherapy combinations.

## 5 CONCLUSIONS

In conclusion, the current study results highlight the possibility of using of a combination of PD‐L1 TPS and circulating CRP as a predictive factor for ICI therapies. Negative predictive markers for ICI therapies are scarce, while they have an important clinical value since most NSCLC patients do not bare treatment benefit. We show that adding peripheral blood CRP value to PD‐L1 score has potential to be an important negative predictive marker for ICI therapies and should be investigated in a prospective fashion.

## AUTHOR CONTRIBUTIONS

**Saara Kuusisalo:** Conceptualization (equal); data curation (equal); formal analysis (equal); funding acquisition (supporting); investigation (equal); methodology (supporting); project administration (supporting); resources (supporting); software (supporting); supervision (supporting); validation (equal); visualization (equal); writing – original draft (equal). **Antti Tikkanen:** Conceptualization (supporting); data curation (supporting); formal analysis (supporting); funding acquisition (supporting); investigation (supporting); methodology (supporting); project administration (supporting); resources (supporting); software (supporting); supervision (supporting); validation (equal); visualization (equal); writing – original draft (equal). **Elisa Lappi‐Blanco:** Conceptualization (supporting); data curation (equal); formal analysis (supporting); funding acquisition (supporting); investigation (equal); methodology (equal); project administration (supporting); resources (equal); software (supporting); supervision (supporting); validation (supporting); visualization (supporting); writing – original draft (supporting). **Timo Väisänen:** Conceptualization (supporting); data curation (equal); formal analysis (supporting); funding acquisition (supporting); investigation (equal); methodology (supporting); project administration (supporting); resources (equal); software (supporting); supervision (supporting); validation (supporting); visualization (supporting); writing – original draft (supporting). **Aija Knuuttila:** Conceptualization (equal); data curation (equal); formal analysis (supporting); funding acquisition (equal); investigation (equal); methodology (equal); project administration (supporting); resources (equal); software (supporting); supervision (supporting); validation (equal); visualization (supporting); writing – original draft (supporting). **Satu Tiainen:** Conceptualization (equal); data curation (equal); formal analysis (equal); funding acquisition (supporting); investigation (equal); methodology (supporting); project administration (supporting); resources (equal); software (supporting); supervision (supporting); validation (equal); visualization (supporting); writing – original draft (supporting). **Jarkko Ahvonen:** Conceptualization (equal); data curation (equal); formal analysis (supporting); funding acquisition (supporting); investigation (equal); methodology (equal); project administration (supporting); resources (equal); software (supporting); supervision (supporting); validation (equal); visualization (supporting); writing – original draft (supporting). **Sanna Iivanainen:** Conceptualization (lead); data curation (lead); formal analysis (lead); funding acquisition (equal); investigation (equal); methodology (equal); project administration (supporting); resources (supporting); software (equal); supervision (equal); validation (lead); visualization (lead); writing – original draft (lead). **Jussi P. Koivunen:** Conceptualization (lead); data curation (lead); formal analysis (lead); funding acquisition (lead); investigation (lead); methodology (lead); project administration (lead); resources (lead); software (equal); supervision (lead); validation (lead); visualization (lead); writing – original draft (lead).

## FUNDING INFORMATION

Study was funded by Oulu University and Finnish Cancer Society.

## ETHICAL APPROVAL AND CONSENT TO PARTICIPATE

The study was approved by the ethics committee of Northern Ostrobothnia Hospital District (study no. 299/2016). Informed consent was not sought due to the register nature of the study

## CONSENT FOR PUBLICATION

Not applicable.

## Data Availability

The datasets generated and/or analyzed during the current study are not publicly available but are available from the corresponding author on reasonable request
